# Glycoconjugate strategy for GLUT-driven curcumin delivery and anticancer activity in breast cancer cells

**DOI:** 10.1038/s41598-026-65680-5

**Published:** 2026-08-14

**Authors:** Safaa S. Hassan, Fatma B. Rashidi, Ramy G. Seddik, Areej W. El-Sayed, Habiba A. Mohamed, Hager W. Mohamed, Menna M. Kenawy, Nouran B. Hussein, Nourhan A. Afify, Noura M. Megahed, Reem M. Zeinhom, Ziad A. Abdultawab, Ayatallah Elgohary, Nashwa Hamido, Ahmed I. Elwakil, Hadeel A. Alzoghaly, Martina O. Nabil, Ehab A. Ahmed, Khaled M. Ismail

**Affiliations:** 1 Chemistry Department, Faculty of Science, Cairo University, Giza, Egypt; 2 Biochemistry Division, Chemistry Department, Faculty of Science, Cairo University, Giza, Egypt; 3 Biotechnology Department, Faculty of Science, Cairo University, Giza, Egypt; 4 Faculty of Biotechnology, October University for Modern Sciences and Arts (MSA), Giza, Egypt; 5 Medical Genome Center, Faculty of Medicine, Cairo University, Cairo, Egypt

**Keywords:** Breast cancer, Curcumin-glycoconjugate, DFT- molecular docking, GLUT receptor, Apoptosis, AMPK–STK11 signaling, Biochemistry, Cancer, Chemical biology, Chemistry, Drug discovery

## Abstract

Selective targeting of cancer cells is a critical strategy in anticancer therapy, and the overexpression of glucose transporters (GLUTs) in malignant cells provides an attractive avenue for achieving this selectivity. In this study, we report the synthesis and biological evaluation of glycoconjugate vanadyl complexes as GLUT-directed anticancer agents against breast cancer. A Schiff base ligand (CG) was synthesized through the condensation of curcumin (C) and glucosamine (G), followed by complexation with vanadyl to form [VO(CG)₂]·5H₂O. The synthesized compounds were comprehensively characterized by elemental analysis, MS, NMR, FT-IR, TGA, molar conductance, and magnetic susceptibility measurements. Spectroscopic and computational studies confirmed that the ligand coordinates in a bidentate chelating mode via the azomethine nitrogen and the enolic oxygen. Biological evaluation in MCF-7 breast cancer cells included cytotoxicity assays, molecular docking, DNA-binding studies, and quantitative gene expression analysis. Both compounds exhibited cytotoxic activity, with the CG ligand showing the highest potency (IC₅₀ = 7.51 ± 0.12 μg/mL or 14.2 μM). Notably, co-treatment with the GLUT inhibitor quercetin significantly increased the IC₅₀ value, suggesting the involvement of GLUT-mediated cellular uptake. Molecular docking studies indicated favorable binding affinity toward GLUT, while DNA-binding experiments demonstrated interaction with DNA. Treatment of MCF-7 cells with the glycoconjugate ligand induced DNA damage, modulated the expression of cell cycle- and apoptosis-related genes, and promoted apoptosis. Flow cytometric analysis demonstrated that the glycoconjugate significantly arrested MCF-7 cells in the S-phase (49.40% vs 22.22% in control). This finding is consistent with impaired cell cycle progression and may be associated with the observed DNA interaction. Quantitative real-time PCR analysis of the CG compound revealed significant upregulation of BAX and CDKN1A (p21) (*p* ≤ 0.05) and pronounced upregulation of STK11 (LKB1) (*p* ≤ 0.001), suggesting activation of the AMPK signaling pathway and a metabolic stress response. Overall, the glycoconjugate ligand induces apoptosis and cell cycle arrest through oxidative and metabolic stress mediated by AMPK–STK11 signaling, highlighting its potential as a GLUT-targeted chemotherapeutic agent for breast cancer treatment.

**Supplementary Information:**

The online version contains supplementary material available at 10.1038/s41598-026-65680-5.

## Introduction

Cancer is recognized as one of the leading causes of mortality worldwide^1^. Lung, prostate, colorectal, stomach, and liver cancers are the most commonly diagnosed cancers in men, whereas breast, colorectal, lung, cervical, and thyroid cancers predominate among women^2^. By 2030, the annual number of new cancer cases and cancer-related deaths is projected to reach 21.4 million and 13.2 million, respectively^3^. Cancer cells are characterized by markedly increased rates of aerobic glycolysis compared with normal cells, resulting in enhanced glucose uptake and accumulation within the cytoplasm^4^. This phenomenon is widely recognized as the “Warburg effect,” which arises from the inefficient ATP production capacity of cancer cells and is a hallmark of cancer^5^. Breast cancer is the second most common cancer diagnosed in women and remains the leading cause of cancer-related mortality among women worldwide^6^.

Curcumin is a polyphenolic compound chemically known as diferuloylmethane. It is the primary bioactive compound in turmeric and is widely used as a natural coloring and flavoring agent in foods^7^. Curcumin has been reported to modulate growth factors, enzymes, transcription factors, kinases, inflammatory cytokines, as well as proapoptotic (by upregulation) and antiapoptotic (by downregulation) proteins. Accordingly, curcumin, alone or in combination with other agents, has been proposed as a promising therapeutic candidate for cancer treatment^8^. Curcumin exhibits anticancer activity across multiple cancer types through mechanisms including inhibition of cell proliferation, suppression of invasion and migration, induction of apoptosis and autophagy, reduction of cancer stemness, increased reactive oxygen species generation, attenuation of inflammation, induction of ferroptosis, modulation of gut microbiota, and application as an adjuvant therapy^9^.

Glucosamine is an amino sugar naturally synthesized in the human body and is widely recognized for its use as a dietary supplement for joint health. However, recent studies have suggested that glucosamine may exhibit anticancer and anti-inflammatory activities, thereby attracting increasing interest in cancer research. Glucose transporters (GLUTs) regulate tumor cell metabolism and represent attractive therapeutic targets for disrupting the energy supply required for cancer cell growth and survival. The combination of curcumin and glucosamine has demonstrated promising outcomes, including improved bioavailability and enhanced therapeutic efficacy of curcumin. This synergistic effect is attributed to the complementary mechanisms of action of both compounds, with glucosamine contributing, in part, through its anti-inflammatory activity, a key factor in cancer progression^10^.

Metal-based compounds exhibit unique physicochemical properties that are often unattainable with organic molecules, including diverse coordination geometries, accessible redox states, and distinct kinetic and thermodynamic behaviors^11^. Vanadium is an essential trace element that plays important roles in various biochemical processes in the human body^12^. Vanadium complexes exhibit a broad spectrum of pharmacological activities, including antidiabetic, antiparasitic, anti-HIV, antitubercular, and anticancer effects, thereby stimulating significant interest in medicinal chemistry research^13–17^. The first vanadyl complexes reported to exhibit anticancer activity against several cell lines were vanadocene dichloride and its derivatives, with IC₅₀ values lower than that of cisplatin^18^.

The selective internalization of glycoconjugate metal complexes increases intracellular metal concentrations, thereby inducing oxidative stress or DNA damage^19^. These effects may subsequently trigger apoptotic pathways, including the activation of pro-apoptotic genes such as BAX^20^. BAX, a member of the BCL-2 protein family, has shown enhanced response to therapy. Agents that induce **BAX** gene expression have been reported to resensitize **TNBC** cells to chemotherapy^21^. Additionally, glycoconjugate complexes are thought to alter cell cycle progression through multiple mechanisms^22^. Glucosamines have been reported to induce cell cycle arrest at the G0/G1 and G2/M phases^23^. This effect was accompanied by increased expression of the cyclin-dependent kinase inhibitor **CDKN1A**, a key mediator of cell cycle arrest. Moreover, some ruthenium(II) glycoconjugates have demonstrated the ability to induce cell arrest at both the G2/M and S phases^22^. These effects suggest modulation of cell cycle regulatory proteins, including cyclin-dependent kinase inhibitors such as CDKN1A and kinases such as CDK6. In addition, serine/threonine kinase 11 (STK11) functions as a tumor suppressor through its role in regulating cellular growth, metabolism, and apoptosis. Loss of STK11 has been reported to enhance the creation of an immunosuppressive tumor microenvironment that supports tumor progression^24^. Only a limited number of studies have reported on vanadium compounds incorporating sugar-containing ligands^4^.

The workflow of this study started with synthesizing a curcumin–glucosamine ligand and then coordinating it to create a VO(II) complex. The cytotoxicity of these compounds was later tested against the MCF-7 breast cancer cell line using the MTT assay, followed by inhibition studies with quercetin as a GLUT inhibitor. Mechanistic studies included evaluating anti-inflammatory activity and analyzing DNA binding through gel electrophoresis, viscosity measurements, and thermal denaturation tests. Protein-binding interactions with bovine serum albumin (BSA) were also examined, along with the impact on key genes related to apoptosis and the cell cycle (BAX, CDK6, CDKN1A, and STK11), using quantitative real-time PCR (qtPCR).

## Materials and methods

All starting chemicals were of high purity and were used without further purification. Curcumin, vanadyl sulfate pentahydrate, quercetin, and glucosamine hydrochloride were purchased from Sigma-Aldrich. All solvents used in the study were of analytical reagent grade. The MCF-7 human breast cancer cell line was obtained from VACSERA, Cairo, Egypt.

### Synthesis of curcumin-glucosamine ligand (CG)

Curcumin (1.0 mmol) was dissolved in 15 mL of methanol and mixed with glucosamine hydrochloride (1.0 mmol), which was previously neutralized using a potassium carbonate solution. The resulting reaction mixture was stirred and heated at 60 °C for 12 h. An orange-brown product was filtered, washed with ethanol, and dried.

### Preparation of Schiff Base VO(II) complex

**Scheme 1:**
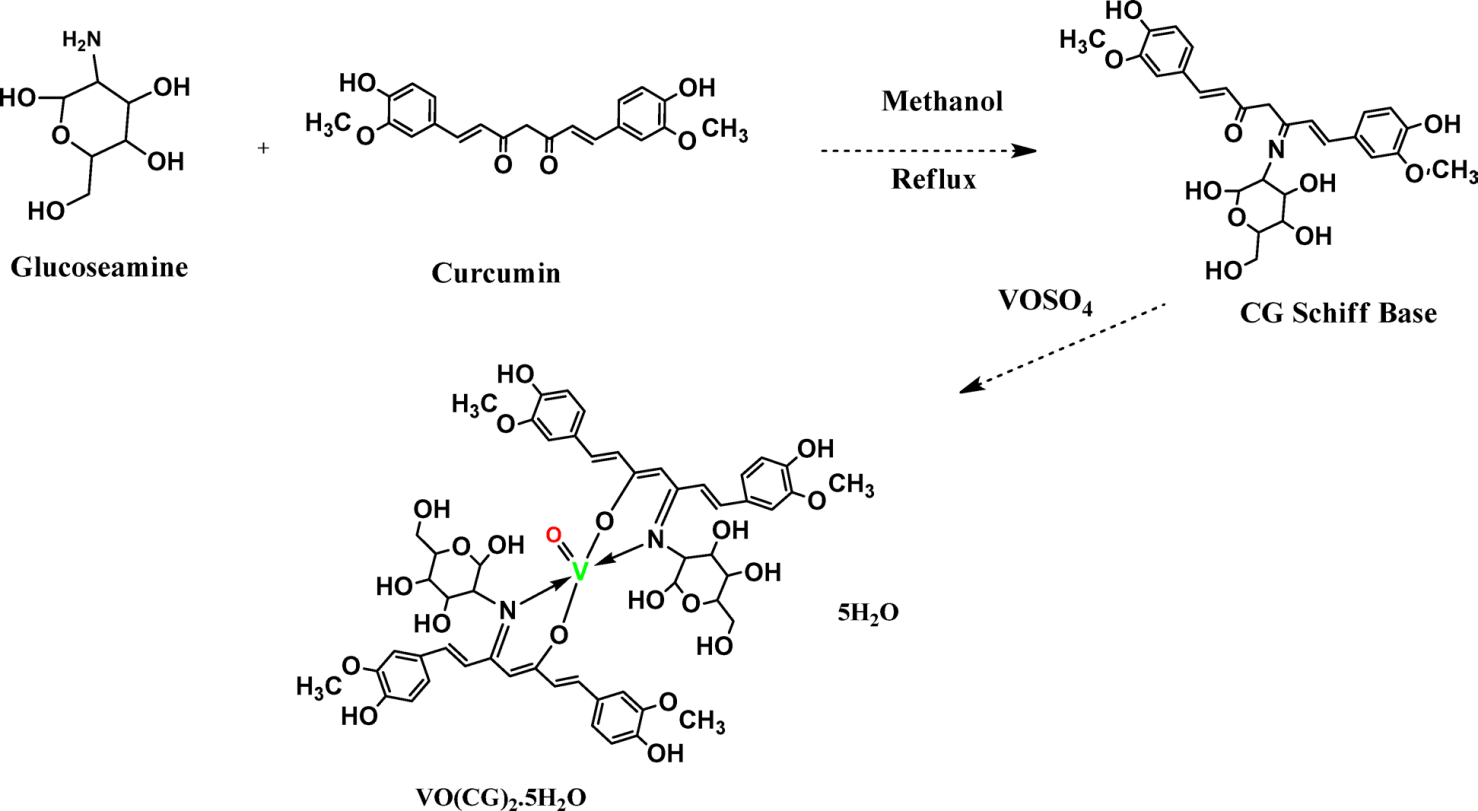
Proposed structures of the CG ligand and its hydrated vanadyl complex, [VO(CG)_2_]‧5H_2_O.

The VO(II) complex was synthesized by reacting VOSO₄·5H₂O with the CG ligand in a 1:2 molar ratio. The reaction was carried out in pure ethanol at 60 °C for about 6 h. A dark brown product was filtered, washed with excess ethanol, and dried. The reaction pathway for the synthesis of the CG ligand and its VO(II) complex is illustrated in Scheme 1.

(C_54_H_70_N_2_O_26_V): Calculated, C, 53.42; H, 5.81; N, 2.31. Found, C, 53.09; H, 5.40; N, 2.28.

### Analytical methods and physical measurements

See the Supplementary Information.

### Cytotoxicity investigation on MCF-7 cell line

See the Supplementary File^25^.

### GLUT inhibitor mediated cytotoxicity assay

Following the cytotoxicity assay, quercetin (90 µg/mL), a GLUT inhibitor^26,27^, was added to MCF-7 cells in combination with varying concentrations of the CG ligand. Cells were incubated for 24 h, and the percentage of cell viability was determined.

### Cell cycle analysis using flow cytometry

To evaluate the effect of the CG ligand on cell cycle distribution, flow cytometric analysis was performed using Propidium Iodide (PI) DNA staining. MCF-7 breast cancer cells were seeded at a density of 1** × **10^5^ cells per well in 6-well plates and incubated at 37 °C with 5% CO₂ for 24 h to allow attachment. After attachment, the cells were treated with the CG ligand at its IC₅₀ concentration for 24 h. Untreated cells in standard media served as controls. After incubation, cells were collected by trypsinization, washed twice with cold phosphate-buffered saline (PBS), and fixed overnight in 70% ice-cold ethanol at -20 °C. Before analysis, the fixed cells were centrifuged, washed with PBS, and resuspended in a staining solution containing 50 μg/mL PI and 100 μg/mL RNase A. Samples were incubated in the dark at room temperature for 30 min. We measured DNA content and cell cycle distribution (G0/G1, S, and G2/M phases) using a BD FACSCalibur flow cytometer. Data were acquired on a logarithmic scale to cover the dynamic range of the assay; however, offline cell-cycle modeling and statistical quantification of cell-cycle phases were performed on the linearized fluorescence parameter using [ModFit LT] to strictly adhere to the linear relationship between DNA content and fluorescence intensity. Notably, the linear supplementary figures were generated using the exact same linearized fluorescence parameter output by the modeling software (ModFit LT) used for the quantitative analysis, ensuring that the G2/G1 peak ratio visually conforms to the expected 2:1 linear relationship.

### Protein denaturation inhibition test

See the Supplementary File^28^.

### DNA binding test (Gel electrophoresis)

DNA gel electrophoresis was used to assess the interaction of the synthesized compounds with DNA by monitoring changes in DNA mobility and band intensity at increasing compound concentrations. Binding of the complexes to DNA may induce conformational changes that affect DNA mobility and band intensity during gel electrophoresis. The assay was conducted in 20 mM Tris–HCl buffer containing 50 mM NaCl (pH 7.3) at a constant CT-DNA concentration of 50 µg/mL, with drug concentrations ranging from 10 to 40 µg/mL. The reaction mixtures were incubated at 37 °C for 2 h. Next, a 1% (w/v) agarose gel was prepared in TBE buffer (45 mM Tris, 45 mM boric acid, and 1 mM EDTA, pH 7.3). The reaction mixture was mixed with 6 × gel-loading dye and then subjected to gel electrophoresis for 45 min at 60 V. The gel was stained with ethidium bromide (EB) for 5 min at room temperature and visualized under a UV transilluminator.

### Viscosity test

To study the effect of the tested compounds on calf thymus (CT-DNA), viscosity measurements were performed at room temperature using an Ostwald viscometer^29^. This experiment aims to provide insight into the binding mode of the tested complex with DNA by monitoring changes in DNA viscosity (and thus DNA effective length). Different concentrations of the tested compounds (0 to 100 μg/mL) were incubated with CT DNA (100 μg/mL) in double-distilled water. The relative viscosity (η) was calculated using the formula η = t/v, where **t** is the flow time and **v** is the volume of the liquid between two fixed points on the viscometer. The relative specific viscosity $(\eta /{\eta}_{0}{)}^\frac{1}{3}$ was then plotted against the ratio of complex to DNA concentration ([complex]/[DNA]) to evaluate binding interactions.

### Thermal denaturation

DNA melting profiles were recorded to evaluate the effect of complex formation on the thermal stability of the CT-DNA^30^. Thermal denaturation experiments were carried out by observing the change in the absorption of CT-DNA at 260 nm at various temperatures to estimate the melting temperature (T_m_). Where T_m_ is the temperature at which 50% of double-stranded DNA becomes single-stranded. T_m_ was measured in the absence and presence of the complexes in a 50 μM Tris–HCl buffer (pH = 7.1) + 5 μM NaCl, which contains a mixture of 50 μM CT-DNA and 50 μM of the complex. The mixture was stirred continuously, and the temperature was increased gradually from 25°C to 90°C, and the reading of absorbance was taken every 5 min. All measurements were performed using a Shimadzu UV spectrophotometer equipped with a TB-85 thermostated cell holder. T_m_ was determined as the mid-point of the hyperchromic transition. All Tm measurements were performed in triplicate, and the results are presented as mean values.

### BSA binding test

See the Supplementary File^31^.

### Total RNA extraction

Total RNA was extracted from the **MCF-7 cell lines** (control and treated) using the TransZol Up kit. Samples were kept on ice during handling to inhibit RNase activity. The concentration and purity were assessed using IMPLEN N50 NanoPhotometer.

### Real-time PCR (qtPCR)

Total mRNA was reverse transcribed into complementary DNA (cDNA) using the (FIREScript® RT cDNA synthesis kit). The gene expression levels of BAX, CDK6, CDKN1, and STK11 were quantified using real-time PCR with the synthesized cDNA. Real-time PCR was performed using SimplyGreen qPCR Master Mix and Rox. For normalization, GAPDH was used as the internal reference. Samples were processed via the StepOne Real-Time PCR System by Thermo Fisher Scientific. All PCR reactions were performed in triplicate to ensure reliability. Relative gene expression levels were calculated using the comparative (ΔΔCt) method.

### qtPCR primers

(Table 1).

**Table 1:** The primer sequences of the studied genes via qt-PCR.

Gene	Forward Primer	Reverse Primer
Bax	GTTTCATCCAGGATCGAGCAG	CATCCTCTGCAGCTCCATGT
CDK6	TTCCCAGGCAGGCTTTTCAT	CTGTATTCAGCTCCGAGGTGTTCT
CDKN1	AGGTGGACCTGGAGACTCTCAG	TCCTCTTGGAGAAGATCAGCCG
STK11	CGCAGCTGAGCACCAAATC	CCTGGACACGGGCTGC
GAPDH	CTTTTGCGTCGCCAGCCGAG	TGAGGTCAATGAAGGGGTCA

Statistical analysis was conducted using SPSS version 25 to determine the significance of the gene expression fold change.

## Results and discussion

### Structural characterization

The results of elemental microanalysis (C, H, and N) confirm the formation of a 1:2 (VO:CG) complex. The experimentally obtained values are consistent with the calculated values based on the proposed molecular formula (Scheme 1). The synthesized compounds are stable under ambient conditions. The non-electrolytic nature of the chelate was confirmed by measuring the molar conductance of a 10^−3^ M solution in DMF^32–34^.

**Fig. 1:**
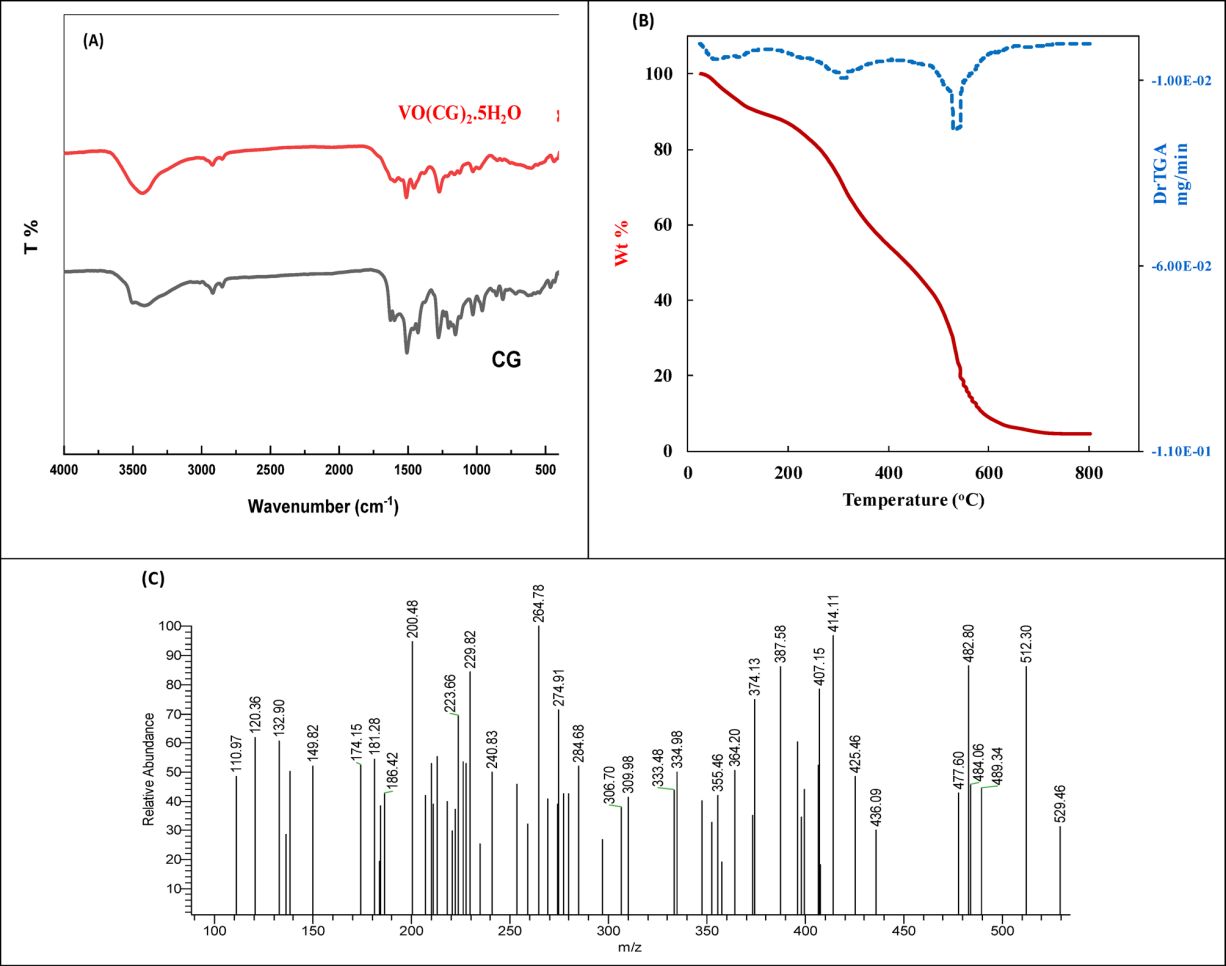
(**A**) FTIR of CG and [VO(CG)_2_]‧5H_2_O complex, (**B**) TG curve of the [VO(CG)_2_]‧5H_2_O, (**C**) Mass spectrum of the CG ligand.

FT-IR Spectral Analysis: The FT-IR spectra, shown in Fig. 1A, provide evidence for the formation of the CG ligand and its vanadyl complex, as well as the proposed structure and coordination mode (Scheme 1). The diagnostic absorption band corresponding to ν(C = O) at approximately 1630 cm⁻^1^, observed in curcumin, disappears in the CG ligand, indicating effective condensation of glucosamine with curcumin and formation of an imine bond. Moreover, the appearance of a new band at 1511 cm⁻^1^, attributed to the ν(C = N) stretching vibration, further confirms the formation of the CG ligand. In vanadyl chelate (Fig. 1A), the band observed at 1506 cm⁻^1^ is assigned to the ν(C = N) stretching vibration, confirming the formation of the complex^35^. This slight shift to lower wavenumber compared with typical azomethine linkages may result from donation of the azomethine nitrogen lone-pair electron density to the metal ion, indicating involvement of the π-electron system in coordination^36,37^. In the infrared spectrum of the complex, the –OH stretching band observed at approximately 3456 cm⁻^1^ shows no significant shift, confirming that the phenolic hydroxyl groups are not involved in coordination with the vanadyl ion^38^. Overlapping of this band with vibrations from hydrated water molecules was also observed^39,40^. The band observed at approximately 1032 cm⁻^1^ may be attributed to δ(C–O) bending vibrations of the phenolic ring^41^. The bands observed at approximately 2920 cm⁻^1^ and 805 cm⁻^1^ are assigned to ν(C–H) stretching and δ(C–H) bending vibrations, respectively^42^. An absorption band observed at 972 cm⁻^1^ in the vanadyl complex is attributed to the ν(V = O) stretching vibration. This observation is consistent with previous reports that assign the ν(V = O) vibration to the region around 972 cm⁻^1^ in similar complexes^4,27^. Weak bands observed at approximately 431 cm⁻^1^ and 596 cm⁻^1^ in the spectrum of the metal complex are attributed to ν(V–N) and ν(V–O) vibrational modes, respectively, providing clear evidence for coordination of nitrogen and oxygen atoms to the metal ion^4,27^. The proposed structure of the complex, illustrated in Scheme 1, represents a hydrated vanadyl Schiff base complex, [VO(CG)_2_]‧5H_2_O. In this structure, the vanadyl ion is coordinated by two CG ligands, each acting as a bidentate chelator through nitrogen and oxygen donor atoms. The presence of water molecules in the hydrated form further stabilizes the coordination complex.

**Thermogravimetric analysis** was used to investigate the thermal stability and decomposition profile of the [VO(CG)_2_]‧5H_2_O complex, as shown in Fig. 1B. The TGA curve reveals three main successive mass loss steps. The initial mass loss occurs at approximately 107 °C and corresponds to the release of five hydrated water molecules, with a mass loss percentage of 6.97% (Exp.) and 7.30% (Theo.). Following dehydration, the compound undergoes two abrupt decomposition phases, which are ascribed to the ligand breakdown with mass loss (Exp. = 88.52% & Theo. = 88.56%). Upon heating up to 800 °C, metallic vanadium was obtained as the final residual, and the calculated and observed residual mass percentages (Exp. = 4.51% & Theo. = 4.13%) agreed well.

**Mass Spectral Analysis:** The mass spectrum of the synthesized CG ligand (Fig. 1C) exhibits a peak at m/z 529.46, corresponding to the molecular ion [M]⁺. These results are in good agreement with the proposed molecular formula of the ligand, as illustrated in Scheme 1.

### DFT study

**Fig. 2:**
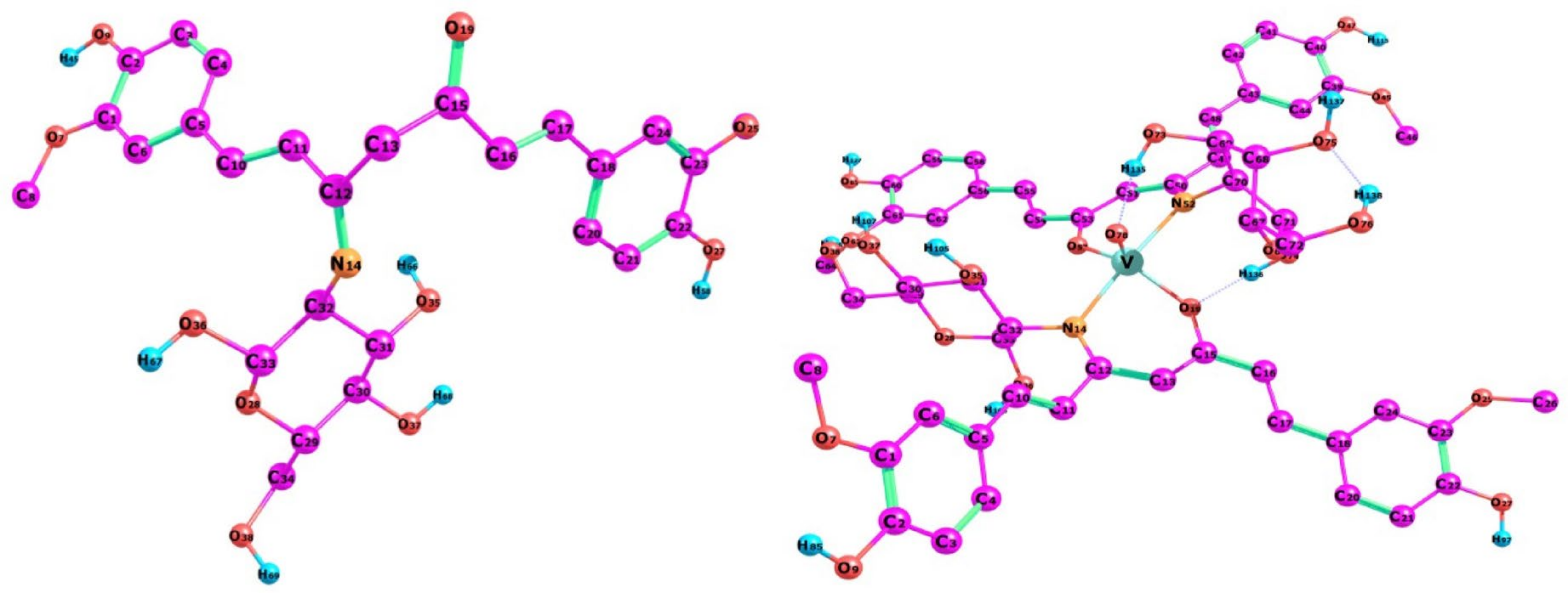
Geometric structure of the CG ligand and the [VO(CG)_2_] complex.

Figure 2 illustrates the optimized structures of the CG ligand and its vanadyl chelate, while Table S1 presents the anticipated bond lengths and angles. Upon coordination with the VO(II) ion, the bond lengths of the CG ligand, particularly those at the donor sites, undergo significant changes. Certain bonds undergo elongation and contraction to achieve the desired coordination structure, as detailed in Table S1. Following coordination, new bonds are formed between the CG ligand (specifically, the enolic oxygen and the nitrogen of the imine group) and the VO(II) ion. Alterations in the chelation surrounding angles specifically for **(O19-C15-C16)**, and **(N14-C12-C13)**, occur as a consequence of the bonding during the complex formation.

The DFT study reveals that the VO(II) chelate exhibits a more negative energy value than the CG ligand, indicating a favorable chelation process. The vanadyl electron density increases from + 2 to 1.147 upon chelation, attributed to charge transfer from the CG ligand to vanadium. The molecular electrostatic potential (MEP) analysis identifies specific structural active sites. Atoms with positive electrostatic potential tend to attach to the regions of negative electrostatic potential, which act as active sites for vanadium ion coordination. The MEP analysis of the vanadyl complex reveals negative electrostatic potential areas at the coordination centers (**N14** and **O19**) and positive potential regions across the framework, as shown in Fig. 2, which enhances its biological activity^43^.

**Fig. 3:**
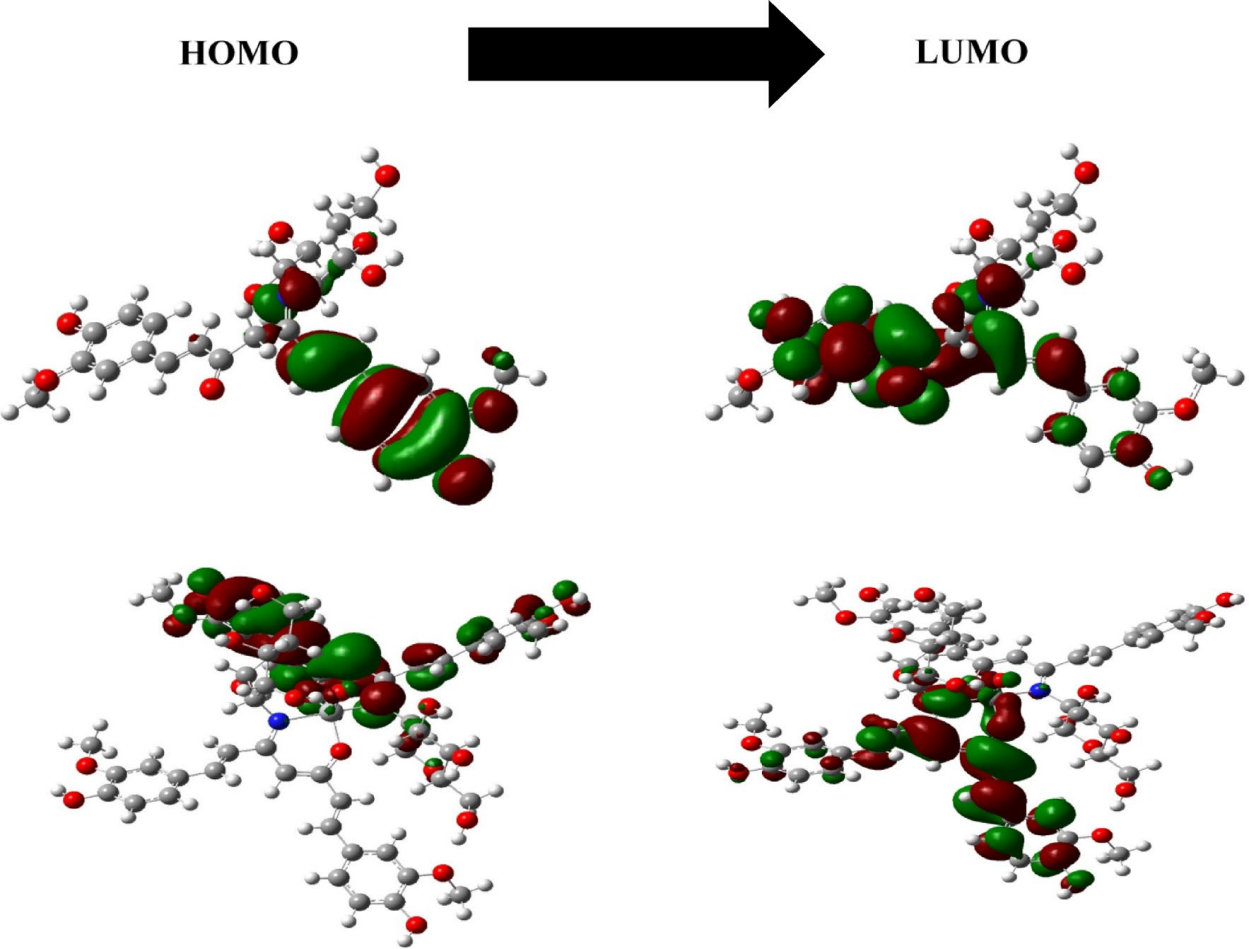
FMO of the CG ligand and the [VO(CG)_2_] complex.

Figure 3 illustrates the exploration of frontier molecular orbitals (FMO). The electron density is concentrated on the heteroatoms, particularly at the donor sites involved in the coordination. Using density functional theory (DFT), several features of the synthesized vanadyl complex, such as dipole moment, hardness (η), softness (S), energy gap, chemical potential (μ), and electronegativity (χ), were calculated and are presented in Table 2. These parameters were computed utilizing the following formulas:

**Table 2:** Ground state properties of the CG ligand and [VO(CG)_2_] chelate, calculated using B3LYP/6-311G and B3LYP/LANL2DZ, respectively.

Parameter	CG	[VO(CG)_2_]	Parameter	CG	[VO(CG)_2_]
E_T_ (Hartree)	− 1854.34	− 3853.92	χ (eV)	4.04	3.575
E_HOMO_ (eV)	− 5.86	− 5.50	η (eV)	− 4.04	0.975
E_LUMO_ (eV)	− 2.22	− 2.60	S (eV^−1^)	0.54	1.025
ΔE (eV)	3.64	1.95	µ (eV)	− 4.04	− 3.57
I = − E_HOMO_ (eV)	5.86	5.50	Dipole moment (Debye)	6.75	8.49
A = − E _LUMO_ (eV)	2.22	2.60			

$${\boldsymbol{\upeta}}=\frac{(\mathrm{I}-\mathrm{A})}{2}, \mathbf{S} =\frac{1}{2\upeta }, {\boldsymbol{\upmu}} =\frac{-\left(\mathrm{I} +\mathrm{A}\right)}{2}, {\boldsymbol{\upchi}} =\frac{(\mathrm{I} + \mathrm{A})}{2}$$

The HOMO–LUMO energy gap reveals that the VO(II) chelate is a softer molecule than its parent CG ligand. As shown in Fig. 3, a smaller HOMO–LUMO gap corresponds to a softer molecule, with the frontier molecular orbitals being represented by the HOMO and LUMO. Hardness is the reverse of softness, which is the capacity to accept electrons^44^. The energy needed to detach an electron is defined by the ionization potential, which corresponds to the energy of the highest occupied molecular orbital (HOMO), while the energy of the lowest unoccupied molecular orbital (LUMO) relates to electron affinity, releasing energy when an additional electron is incorporated into the system. The considerable energy difference between the HOMO–LUMO values of the CG ligand and its chelate illustrates the stability of the molecules. Nonetheless, the VO(II) complex demonstrates heightened susceptibility to electron transfer from electron-rich species. The polarity of the CG ligand increases upon interaction with the VO(II) ion, as evidenced by the larger dipole moment. After chelation, the electron clouds of the FMO orbitals extended to include the metal’s coordination center.

### Measurement of cytotoxicity on MCF-7 cells using a GLUT inhibitor mediated cytotoxicity assay

**Fig. 4:**
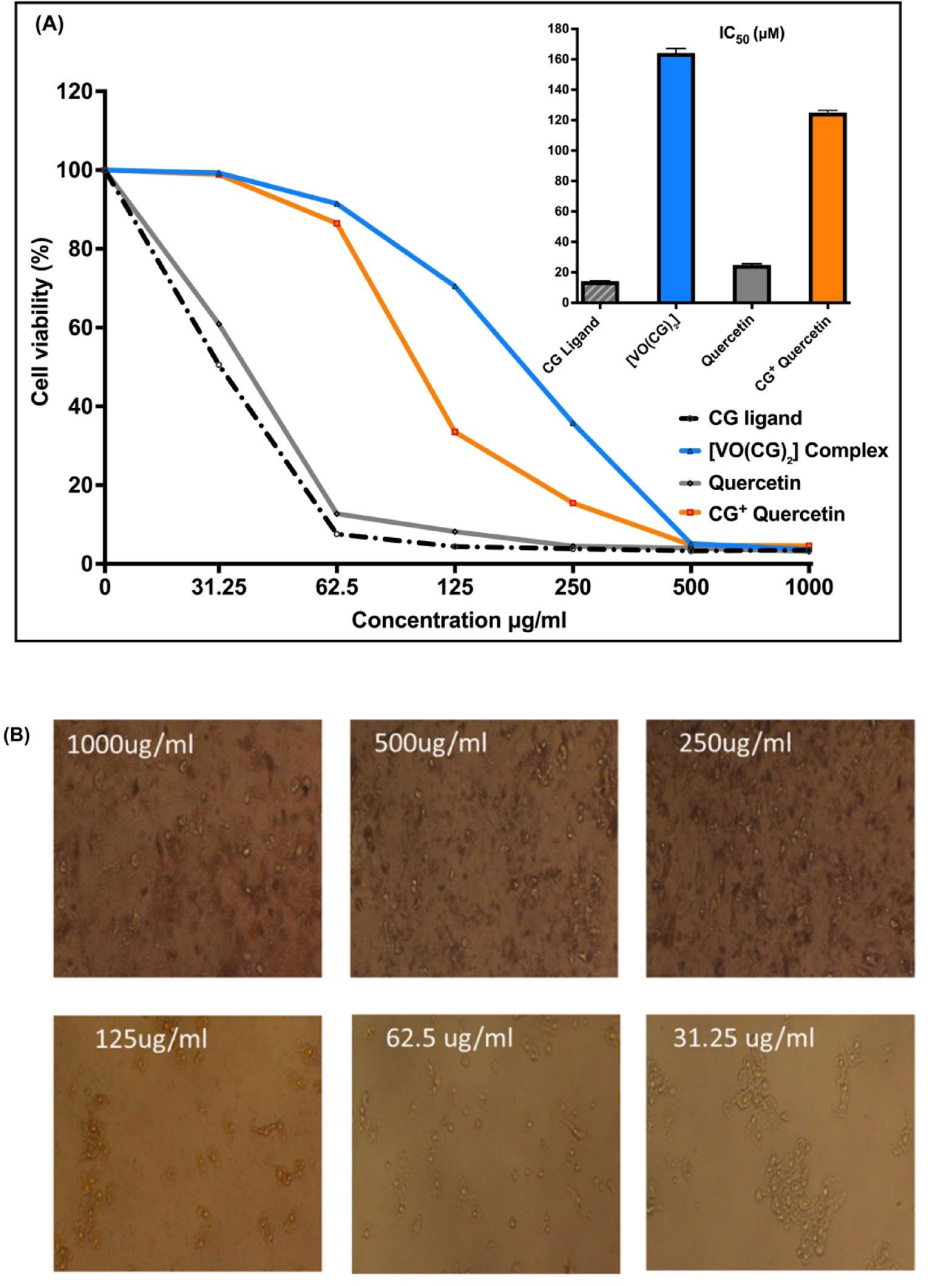

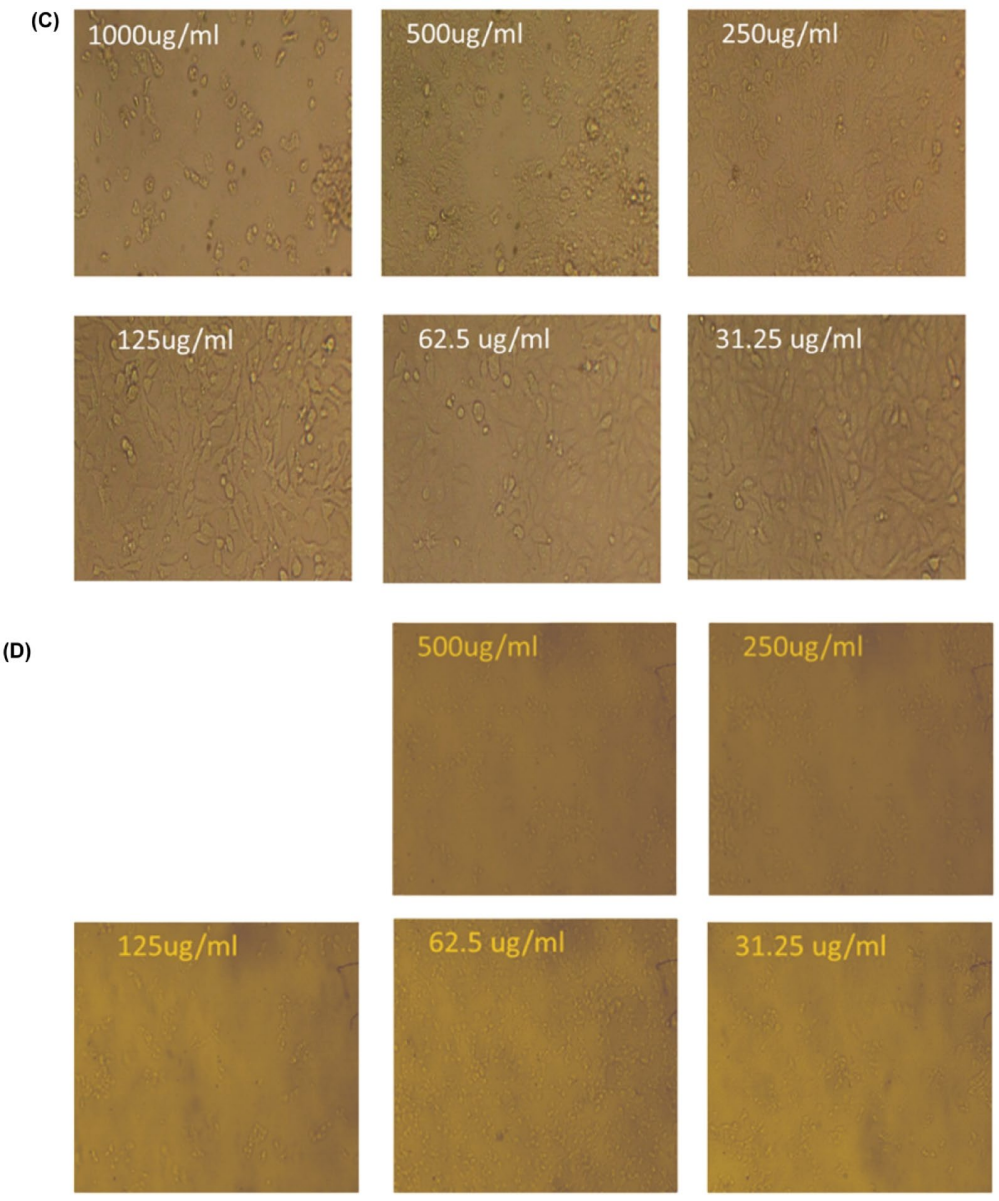

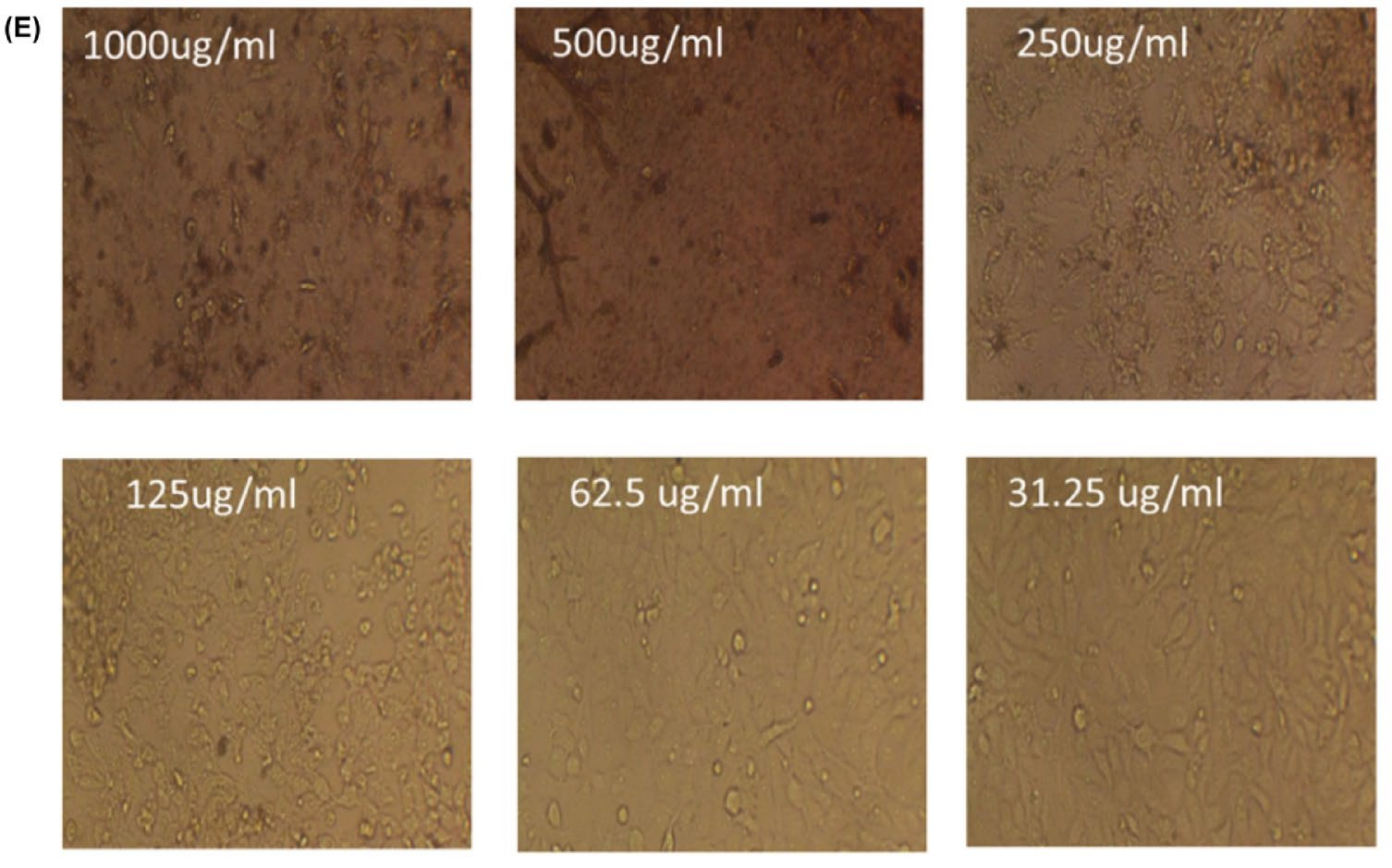
Cytotoxic effects and morphological analysis of MCF-7 breast cancer cells. (A) The MTT assay demonstrated a significant reduction in the viability of the breast cancer cell line (MCF-7) after 24 h of treatment with different concentrations of the free CG ligand, [VO(CG)₂] complex, quercetin, and CG+ quercetin with corresponding IC₅₀ values. (**B**–**E**) Representative inverted microscopy images (60× magnification) illustrating morphological changes in MCF-7 cells after treatment with (B) CG, (C) [VO(CG)₂] complex, (D) Quercetin, and (E) CG + quercetin.

The cytotoxic activity of the synthesized curcumin–glucosamine conjugate (CG) and its vanadyl complex, [VO(CG)₂]·5H₂O, was evaluated against MCF-7 human breast cancer cells using the MTT assay after 24 h of exposure. Both compounds induced a concentration-dependent reduction in cell viability relative to untreated controls (Fig. 4A), and the mean values with their standard deviations are presented in Table S2. The free CG ligand demonstrated significantly higher antiproliferative activity, exhibiting an IC₅₀ value of 7.51 ± 0.12 μg/mL (14.2 μM), whereas the corresponding vanadyl complex showed weak cytotoxicity with an IC₅₀ value of 199.82 ± 3.52 μg/mL (164.3 ± 2.9 μM).

Morphological examination of treated cells under an inverted microscope revealed cellular changes consistent with apoptosis, including reduced cell density, cellular shrinkage, membrane blebbing, and apoptotic body formation (Figs. 4B and C), consistent with the cytotoxic effects observed in the viability assay.

To investigate the potential involvement of glucose transporters (GLUTs) in the cellular uptake of the glycoconjugate, a GLUT inhibition assay was performed using quercetin, a reported inhibitor of GLUT1, GLUT3, and GLUT4. Consistent with previous reports, quercetin alone exhibited marked antiproliferative activity against MCF-7 cells, with an IC₅₀ value of 7.52 ± 0.75 μg/mL (24.9 ± 2.5 μM), reflecting its intrinsic pro-apoptotic and growth-inhibitory properties in breast cancer cells^45^. The concentration of quercetin used for co-treatment (90 µg/mL) was selected based on previously reported concentrations used in cellular studies investigating its GLUT inhibitory activity and biological effects^4,26,46^.

Notably, co-treatment of MCF-7 cells with quercetin at a fixed concentration of 90 µg/mL and varying concentrations of the CG ligand resulted in a significant increase in the IC₅₀ value of CG to 104.11 ± 1.49 μg/mL (125.0 μM), representing a substantial reduction in antiproliferative potency. This reduction in activity is consistent with the possibility that GLUT inhibition may contribute to reduced cellular uptake of the glycoconjugate. The observed antagonistic interaction supports the potential involvement of glucose transporters in modulating CG cellular uptake. These findings suggest that GLUT-mediated uptake may partially contribute to the anticancer activity of CG.

Morphological assessment of cells treated with CG in the presence of quercetin further demonstrated apoptotic features, including decreased cell density, cellular shrinkage, membrane blebbing, echinoid spike formation, and apoptotic bodies (Fig. 4D). Collectively, these findings support a possible contribution of glucose transporters to the cellular uptake of the curcumin–glucosamine conjugate and are consistent with the proposed involvement of GLUT-mediated uptake pathways.

### Apoptotic gene induction

**Fig. 5:**
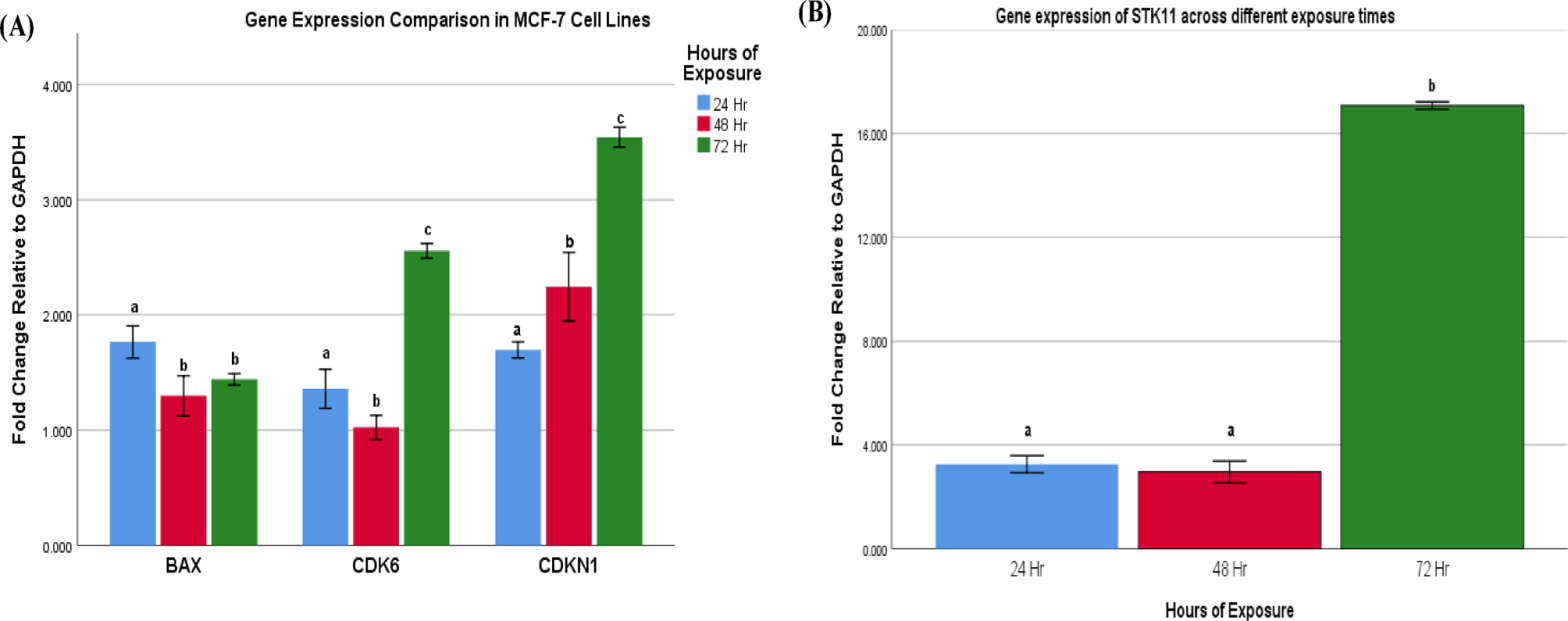
(**A**) Gene expression levels of BAX, CDK6, and CDKN1A in MCF-7 cell lines after 24, 48, and 72 h of CG exposure. Data are shown as fold changes relative to GAPDH and are presented as mean ± SD. Different lowercase letters (a, b, c) indicate statistically significant differences at *p* ≤ 0.05. As “a” any treatment time point (e.g., 24 h) indicates that no significantly different from the control, if the 24 h exposure is significantly different from the control, it gets a "b". If the 48 h exposure is significantly different from both the control and the 24h exposure, it gets a "c". If the 48h exposure is different from the control but the same as the 24 h exposure, it shares the "b". (**B**) Gene expression levels of STK11 in MCF-7 cell lines after 24, 48, and 72 h of exposure. Data are shown as fold changes relative to GAPDH and are presented as mean ± SD. Different lowercase letters (a, b, c) indicate statistically significant differences at *p* ≤ 0.05.

The mRNA expression levels of apoptotic genes such as Bax, alongside cell cycle regulators such as cyclin-dependent kinases CDK6, CDKN1A, and STK11, were measured. All samples were compared to untreated MCF-7 (control) samples. As shown in Fig. 5A, there were significant differences between the different exposure times. When compared to the control, all BAX groups showed a statistically significant upregulation (p ≤ 0.05) across all exposure times. CDK6 gene expression after 24 and 72 h showed a significant upregulation (p ≤ 0.05) compared to the control. In addition, CDKN1A gene expression showed a significant upregulation (p ≤ 0.001) across all exposure times. As shown in Fig. 5B, STK11 showed a significant upregulation (p ≤ 0.001) when compared to control samples.

BAX is a pro-apoptotic gene that promotes apoptosis in cancer cells. Glycoconjugation allowed the free CG ligand to be selectively taken up by MCF-7 cells^47^. The uptake induced DNA damage through the binding of free CG ligand to the DNA. Such genotoxic stress activated cellular apoptotic pathways, including the mitochondrial pathway, by upregulating the expression of the BAX gene. As shown in the results, 24 h of exposure was optimum for elevating the BAX gene expression to activate the apoptotic pathways, which facilitates the activation of the caspase cascade^48^. Additionally, the DNA damage induced by binding of the CG ligand triggered a significant upregulation of the CDKN1A gene. Because CDKN1A encodes the p21 protein—a well-established cyclin-dependent kinase inhibitor—its transcriptional activation suggests a potential molecular mechanism for altering cell cycle progression. However, we acknowledge that mRNA expression levels alone do not definitively confirm functional cell cycle arrest. Further functional investigations, such as flow cytometry analysis, are required to confirm cell cycle arrest and identify any specific phase blocks^49^. In contrast, the upregulated CDK6 may indicate resistance to metabolic and oxidative stress caused by the uptake of the free CG by MCF-7 through GLUT receptors. After 48 h of exposure, the expression of CDK6 was unchanged compared to the control group, while after 72 h of exposure, CDK6 showed upregulation, which might indicate that prolonged exposure to the free CG could lead to cellular resistance to treatment^50^. Moreover, such cellular stress could activate the AMPK pathway. Alterations in AMPK levels further promote the expression of STK11 due to its critical role in regulating cellular energy by negatively regulating the mTOR pathway, which reduces protein synthesis and cell proliferation^51,52^.

While our study evaluated the gene expression profile of the fully synthesized CG glycoconjugate, the individual precursor moieties are well-documented in the literature to independently modulate key cell cycle and apoptotic regulators. Curcumin has been established as a potent modulator of both pro- and anti-apoptotic proteins^8,9^; it targets the AMPK/STK11 signaling axis, upregulates the pro-apoptotic BAX protein, and directly induces cell cycle arrest via the profound upregulation of CDKN1A (p21) in MCF-7 cells^9,49^. Similarly, glucosamine exhibits inherent antineoplastic properties by interfering with tumor metabolism; it induces endoplasmic reticulum stress-mediated apoptosis and triggers cell cycle arrest accompanied by elevated CDKN1A expression^23^.

### Cell cycle analysis using flow cytometry

**Fig. 6:**
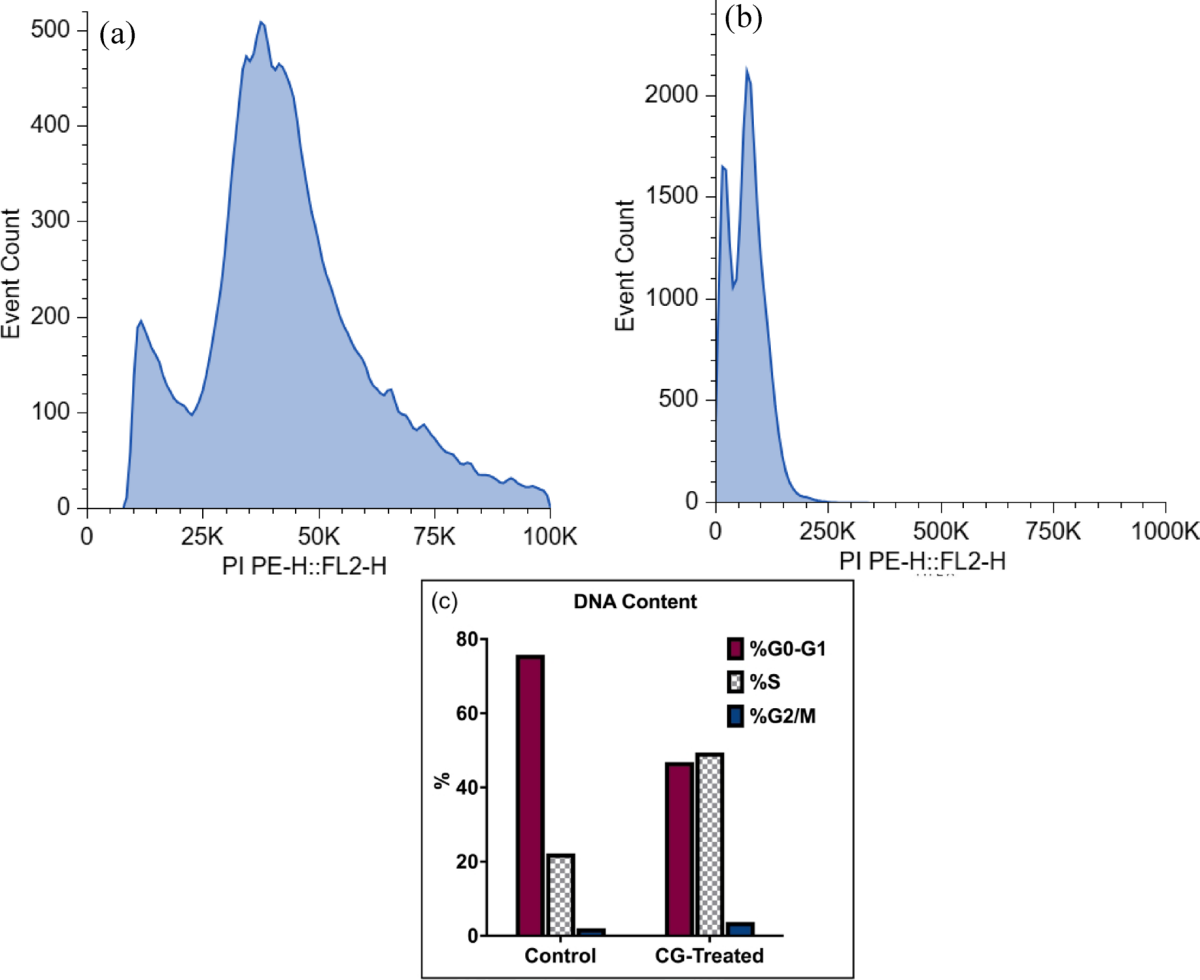
Flow cytometric cell-cycle analysis of MCF-7 breast cancer cells following treatment with the curcumin–glucosamine (CG) ligand. Representative Propidium Iodide (PI) DNA content histograms displayed on a linear fluorescence intensity scale are shown for (**a**) untreated control cells and (**b**) cells treated with the CG ligand at its IC₅₀ (7.51 ± 0.12 µg/mL) for 24 h. (**c**) Distribution of cells in the G0/G1, S, and G2/M phases. Treatment with the CG ligand increased the S-phase population from 22.22 to 49.40%, indicating S-phase cell-cycle arrest.

To better understand the mechanism underlying the antiproliferative activity of the CG ligand, cell-cycle progression in MCF-7 cells was analyzed by flow cytometry following Propidium Iodide (PI) staining. Representative DNA content histograms are presented in Fig. 6 using a linear fluorescence intensity scale, whereas the corresponding logarithmic histograms are provided in Fig. S2 for comparison and improved visualization of the sub-G1 apoptotic population. As shown in Fig. 6, untreated control cells exhibited a typical cell-cycle distribution, with 75.75% of cells in the G0/G1 phase, 22.22% in the S phase, and 2.04% in the G2/M phase. Following 24 h of treatment with the CG ligand, the cell-cycle profile was markedly altered, with the S-phase population increasing to 49.40% and the G0/G1 population decreasing to 46.86%, while only a small proportion of cells remained in the G2/M phase (3.73%). These findings indicate that the CG ligand induces S-phase cell-cycle arrest, thereby impairing normal cell-cycle progression. The observed S-phase arrest is consistent with our DNA-binding and gene expression findings, which together support the antiproliferative activity of the CG ligand. In particular, qPCR analysis revealed significant upregulation of the cyclin-dependent kinase inhibitor CDKN1A (p21) following treatment. Collectively, these findings suggest that disruption of cell-cycle progression may contribute to the induction of apoptosis, consistent with the increased expression of the pro-apoptotic BAX gene and activation of the AMPK–STK11 signaling pathway.

### Thermal denaturation

**Fig. 7:**
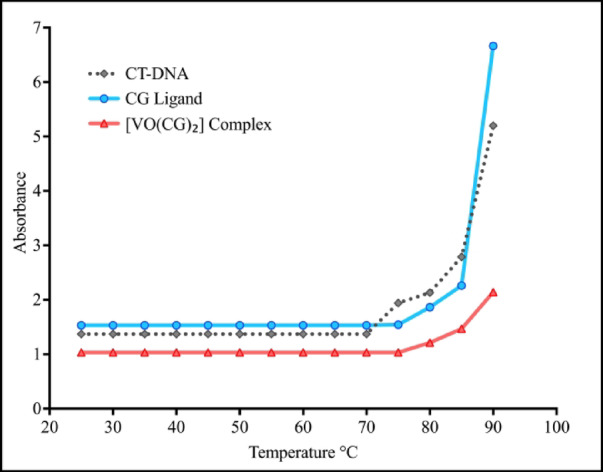
Melting curves of CT-DNA in Tris–HCl Buffer in the absence and presence of the CG ligand and [VO(CG)_2_] complex, [DNA] = 50 μM, [compound] = 50 μM, showing an increase in T_m_ due to Binding.

Thermal denaturation studies were employed to evaluate the effects of curcumin and its vanadyl complex on CT-DNA stability and to infer the nature and strength of their binding interactions^53^. Changes in DNA melting temperature (T_m_) reflect the binding strength of small molecules to DNA^54^, where T_m_ corresponds to the temperature at which half of the DNA duplex is denatured^55^. The melting curves of CT-DNA in the absence and presence of the ligand and complex are shown in Fig. 7. Free CT-DNA exhibited cooperative melting between 75–85 °C, with a T_m_ of approximately 75 °C, consistent with previous reports^56^. In the presence of the ligand and the vanadyl complex, a moderate increase in T_m_ (ΔT_m_ ≈ 5 °C) was observed, indicating stabilization of the DNA duplex.

Although this assay did not include an internal positive control, the thermal denaturation characteristics of traditional intercalators under similar buffer conditions are well-documented. For example, classical intercalators like ethidium bromide often produce notable melting temperature shifts (ΔT_m_) of 10 °C to 15 °C or more, owing to their strong stabilization of the DNA double helix^57^. Conversely, the observed moderate increase (ΔT_m_ ≈ 5 °C) for the vanadyl complex and ligand is considerably lower than these typical intercalative effects. This moderate stabilization is consistent with reports of non-intercalative binding modes, such as groove or electrostatic interactions, which are likely responsible for their primary mode of binding^58^.

The increased thermal stability may also arise from interactions of cationic species with the DNA phosphate backbone^59^. Overall, the thermal denaturation results support a non-intercalative binding mode, most likely groove or electrostatic binding, for the vanadyl complex and ligand with CT-DNA^60^.

### BSA binding studies

Evaluating drug-protein interactions is vital for understanding the pharmacokinetics of new chemotherapeutics. Bovine serum albumin (BSA), similar to human serum albumin (HSA), the main blood carrier for metal drugs, helps assess how well compounds bind. This reveals their transport efficiency, circulation half-life, and bioavailability prior to cellular uptake via GLUT transporters in MCF-7 cells.

**Fig. 8:**
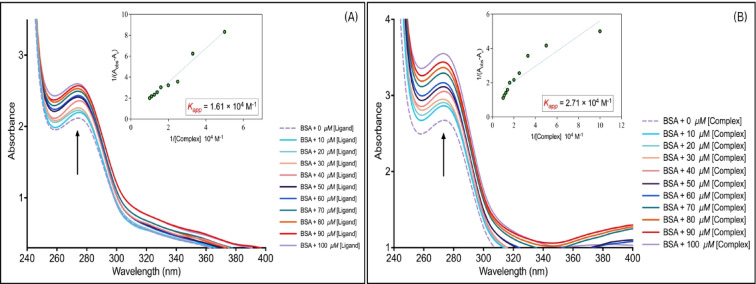
(**A**) UV–visible absorption spectra of the ligand and (**B**) its vanadyl complex with increasing concentrations (10–100 µM) at a constant 1% of BSA in phosphate-buffered saline (PBS). Inset: Linear plots of 1/(A_obs_ − A_₀_) vs. 1/[complex] (10^4^ M) for the binding of BSA to the ligand and vanadyl complex.

The interaction of BSA with the ligand and its vanadyl complex was investigated by UV–Vis absorption spectroscopy. Increasing concentrations of the ligand (10–100 µM) led to a concentration-dependent increase in BSA absorbance at ~ 280 nm, indicating complex formation and changes in the microenvironment of aromatic residues (Fig. 8A). The apparent binding constant (K_app_) was calculated as 1.61 × 104 M⁻1 and 2.71 × 104 M⁻1 for the BSA–ligand system and the BSA–vanadyl complex, respectively (Fig. 8B). These magnitudes suggest a moderate binding affinity and are consistent with values reported for various small organic and metal-based ligands interacting with BSA via non-covalent forces. UV–Vis spectral titration approaches, similar to those used here, have been established in the literature to estimate binding constants by monitoring changes in absorption intensity as ligand concentration increases^61^.

### DNA binding test

**Fig. 9:**
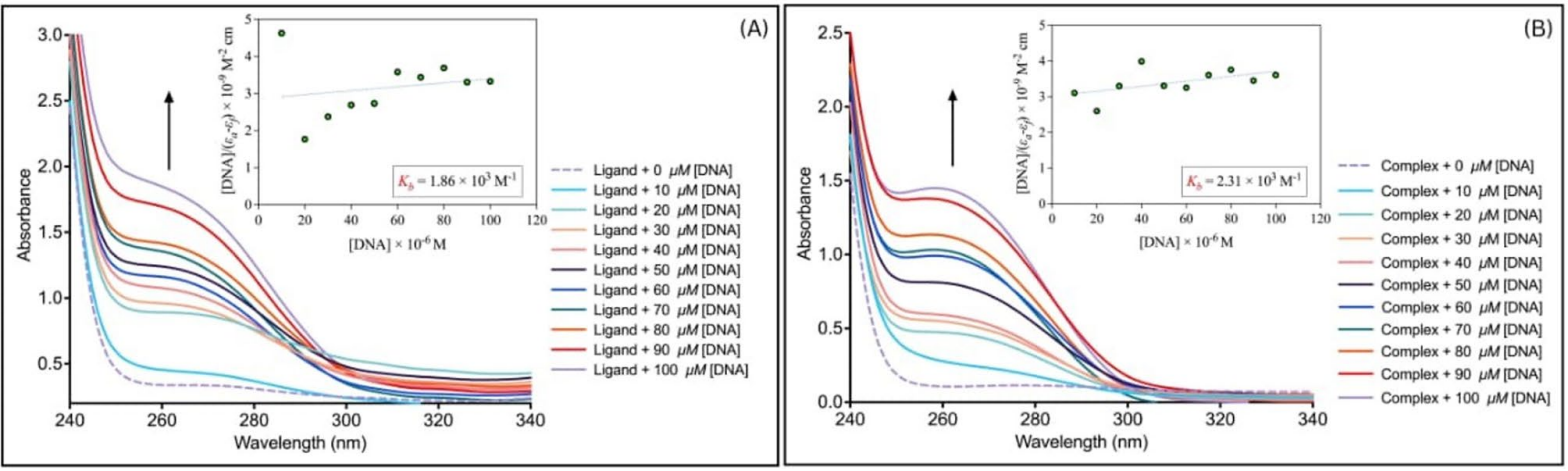
UV–visible absorption spectra of the CG ligand and the VO(II) complex and the ligand at a concentration of 200 μg/mL and 0.2 μg/mL, respectively, following titration with increasing CT-DNA concentrations (0.00–100.00 μM) in Tris–HCl buffer solution. Inset: Linear plots of [DNA]/(Ɛ_a_–Ɛ_f_) × 10^9^ M^2^cm vs. [DNA] for CT-DNA titration to the ligand (**A**) and the [VO(CG)_2_] complex (**B**).

Electronic absorption spectroscopy is a powerful tool for investigating the binding of metal complexes to DNA, where changes in absorption intensity indicate DNA interaction^54^. UV–Vis titration experiments were carried out at fixed concentrations of the vanadyl complex (200 µg/mL) and ligand (0.2 µg/mL), while the CT-DNA concentration was gradually increased from 0 to 100 µM. Upon DNA addition, both the ligand and the vanadyl complex exhibited hyperchromic effects accompanied by a slight red shift in the absorption bands (260–270 nm), suggesting interaction with DNA and partial disruption of the duplex structure (Fig. 9A)^62^. Such spectral behavior is characteristic of intercalative or groove binding modes^53^. The pronounced hyperchromism is attributed to π–π* interactions and possible hydrogen bonding between DNA bases and heteroatom-containing groups of the compounds^63^. The intrinsic DNA-binding constants (K_b_) were calculated from absorption titration data, yielding values of 1.86 × 103 M⁻1 for the ligand and 2.31 × 103 M⁻1 for the vanadyl complex, indicating significant DNA affinity, with the vanadyl complex showing stronger binding ability (Fig. 9B)^64^.

### DNA binding test (gel electrophoresis) and CT-DNA Viscosity

The interaction of the tested vanadyl complex with CT-DNA was studied using agarose gel electrophoresis and viscosity measurements, as shown in Figs. S2 and 10. Gel electrophoresis showed reduced DNA mobility and band intensity with increasing complex concentration, indicating DNA binding and possible conformational changes that limit ethidium bromide intercalation. Viscosity studies revealed only a slight increase in DNA viscosity, suggesting minor structural stiffening without significant helix elongation. Together, these results indicate that the vanadyl complex binds to CT-DNA mainly through groove binding or electrostatic interactions rather than classical intercalation.

**Fig. 10:**
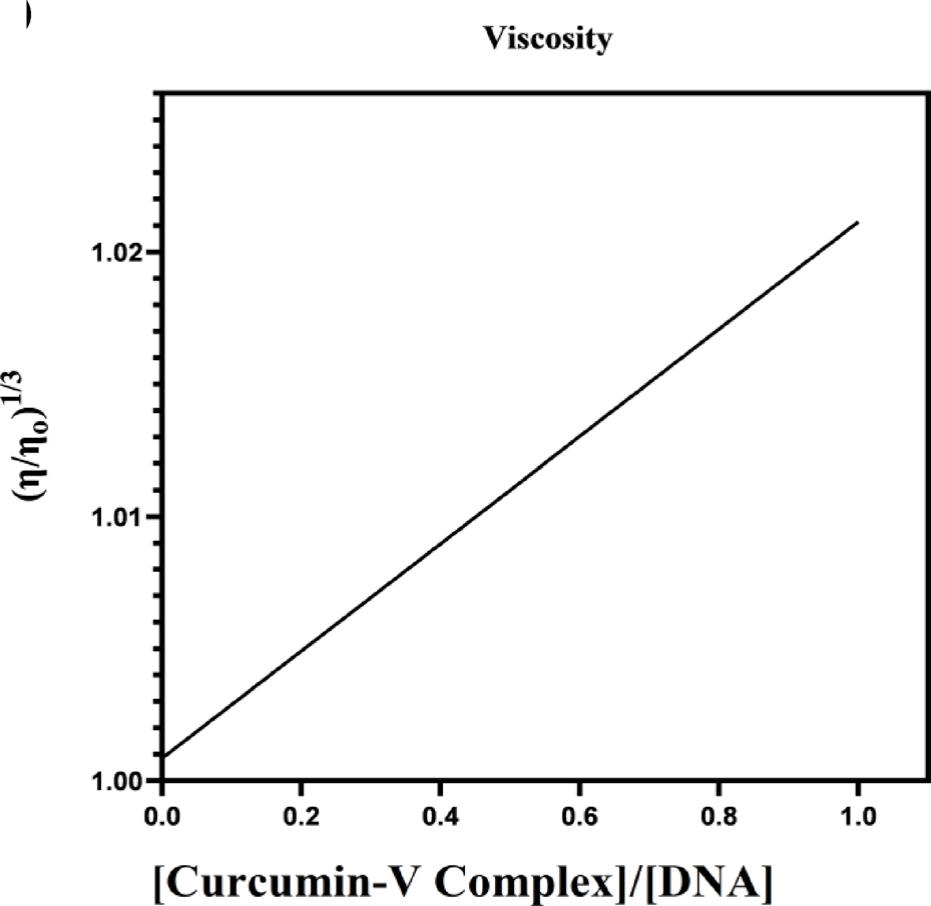
Effect of increasing concentrations of the complex on CT-DNA viscosity. [Complex]/[DNA] = 0.20–1.00, [CT-DNA] = 100 μM.

### Protein denaturation inhibition test

Non-steroidal anti-inflammatory drugs exert their effects mainly through the inhibition of protein denaturation, lysosomal enzyme activity, or membrane stabilization. Inflammation is commonly associated with protein denaturation or lysosomal enzyme release by leukocytes, as observed in arthritis^65^. The prepared CG compound exhibited 50% ± 0.02% inhibition of protein denaturation at 10 µg/mL, indicating its potential as a promising anti-inflammatory agent; however, further studies are required to confirm this activity.

### Molecular docking

#### Docking on EGFR, PTP1B, and BSA receptors

**Fig. 11:**
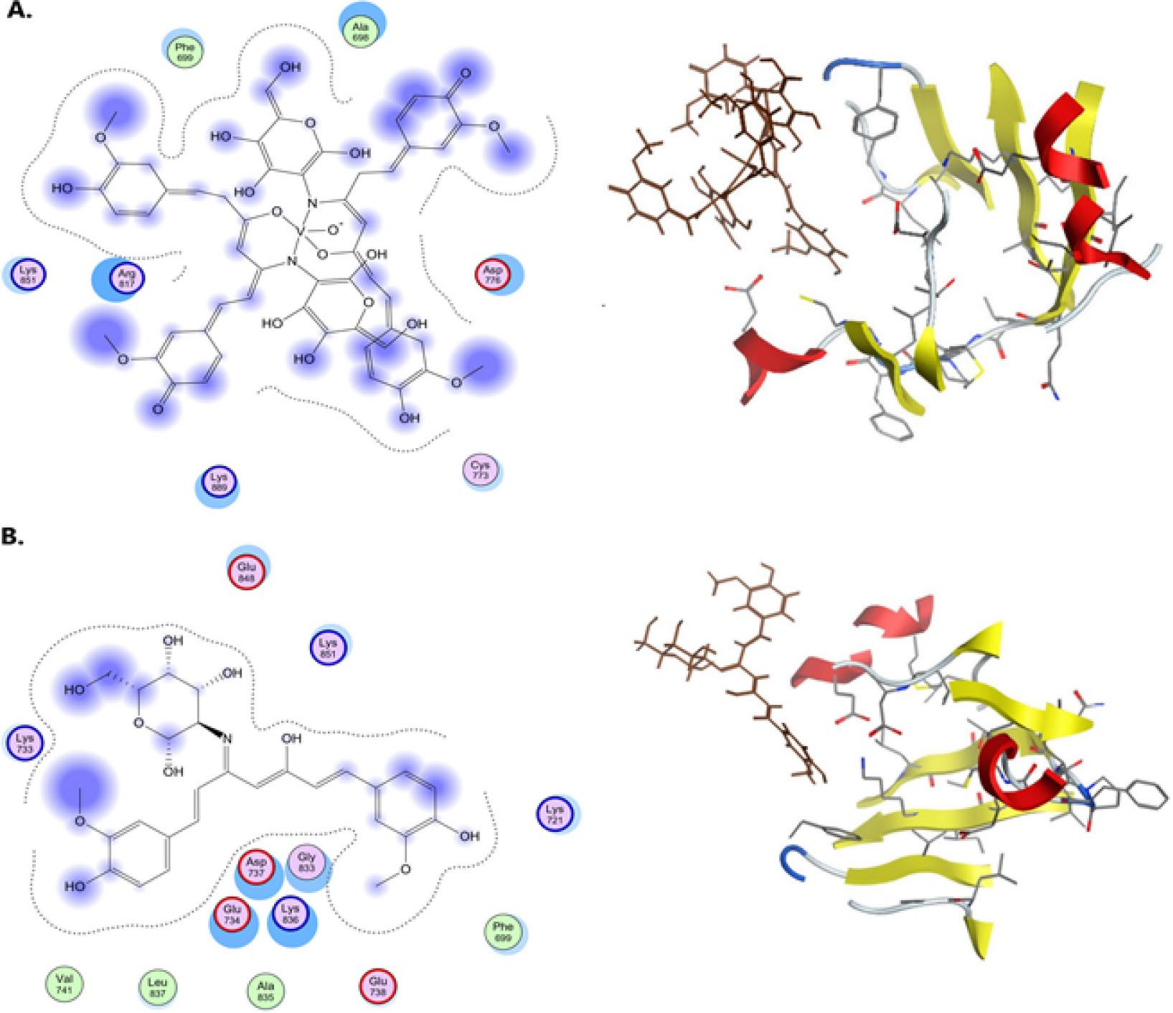
2D and 3D interactions of (**A**) the CG ligand and (**B**) [VO(CG)_2_] complex with the EGFR receptor.

The molecular docking study evaluated the binding interactions of the CG ligand and its vanadyl(II) complex with the EGFR active site (PDB ID: 1M17) to understand their cytotoxic behavior. The ligand showed strong interactions through hydrophobic, hydrogen bonding, and electrostatic interactions with key residues, including Ala698, Arg817, Asp737, Lys836, Glu734, and Gly833 as seen in Fig. 11 and Table S3. These interactions indicate a more stable and stronger binding affinity for the ligand compared to the vanadyl(II) complex.

GLUT receptors, particularly GLUT1, play a crucial role in glucose uptake and are frequently overexpressed in cancer cells to satisfy their elevated metabolic demands. Consequently, GLUT1 has emerged as an attractive molecular target for anticancer drug design. In this context, the affinity of our glycoconjugate compounds toward GLUT receptors, especially GLUT1, was investigated to evaluate their potential targeting capability. The interaction with GLUT1 may facilitate selective uptake of these compounds by cancer cells, thereby enhancing their cytotoxic efficacy through interference with glucose transport and cancer cell metabolism, as seen in Fig. S3 and Table S4.

### ADMET prediction

ADMET predictions using SwissADME showed that both compounds are non-mutagenic, non-hepatotoxic, and non-skin sensitizers, indicating good biocompatibility, as seen in Table S5. The ligand exhibited higher GI absorption, no hERG inhibition, and a higher LD₅₀ value, suggesting better pharmacokinetic behavior and lower acute toxicity than the vanadyl complex. In contrast, the vanadyl complex showed improved chronic tolerance based on its LOAEL value. Overall, the ligand demonstrated a more favorable ADMET profile, while the vanadyl complex may be more suitable for chronic administration under controlled cardiotoxicity conditions.

## Conclusion

In conclusion, glucose moiety–based targeted anticancer therapy demonstrated significant potential in this study. The Schiff base derived from curcumin and glucosamine, along with its vanadyl(II) complex, was successfully synthesized and structurally confirmed through combined theoretical and experimental approaches. Both compounds exhibited notable anticancer activity against breast cancer cells, with targeting specificity mediated through GLUT receptors, as evidenced by the reduced cytotoxic efficacy in the presence of the GLUT inhibitor quercetin. These findings suggest that the glycoconjugated ligand is specifically recognized by the glucose-binding site of GLUT transporters. Mechanistically, the ligand induced profound S-phase cell cycle arrest and apoptosis via oxidative and metabolic stress pathways involving AMPK–STK11 signaling. Additionally, DNA interaction studies and molecular docking analyses supported the ability of the ligand and its vanadyl complex to bind effectively to biological targets, thereby inhibiting protein function. Collectively, these results highlight the promise of sugar-conjugated vanadyl complexes as targeted anticancer agents and provide a strong foundation for further preclinical development.

*“This manuscript represents the outcome of a graduation project conducted by undergraduate students of the Biotechnology program, Chemistry Department, Faculty of Science, Cairo University, and Faculty of Biotechnology, October University for Modern Sciences and Arts.”*

## Supplementary Information

Supplementary Information.

## Data Availability

All data generated or analysed during this study are included in this published article [and its supplementary information files].
