# Supplementary material for: Glycoconjugate strategy for GLUT-driven curcumin delivery and anticancer activity in breast cancer cells
Source: Sci Rep. 2026 Aug 14;16:25478. doi: 10.1038/s41598-026-65680-5 (PMC13476261; doi:10.1038/s41598-026-65680-5)
Supplement: Supplementary file 1 [file 41598_2026_65680_MOESM1_ESM.docx]

**Analytical Methods and Physical Measurements**

Elemental composition was determined using an automatic CHNS analyzer (Vario EL III-Elementary, Germany). Magnetic behavior and electrical conductance were measured using a Scientific balance and a conductivity meter (ORION model 150), respectively. The compounds were characterized using a Shimadzu FTIR spectrometer and a Shimadzu TG 50 thermogravimetric analyzer. An automated UV/Vis–NIR 3101 PC Shimadzu spectrophotometer was used to measure electronic absorption spectra in DMF over the range of 250–900 nm.

**Cytotoxicity Investigation on MCF-7 Cell Line**

An assay employing 3-(4,5-dimethylthiazolyl)2,5-diphenyl-tetrazolium bromide (MTT) was used to assess the cytotoxic impact of the investigated substances [25]. Briefly, 10^4^ cells/100 µl of full RPMI medium/well were used to inoculate MCF-7 cells in a flat-bottomed 96-well plate, and the cells were left to form a finished monolayer sheet overnight. A two-fold dilution series of tested compounds, ranging from 0 to 1000 µg/ml, was created by dissolving the appropriate weight of the tested sample in 1 ml of PRMI medium containing 2% (FBS). The cell monolayer sheet had been washed twice with wash media after 24 hours. Three wells containing inoculum cells received culture medium and acted as the untreated groups. The plate was incubated for 24 hours in a humidified incubator (37 ºC, 5% CO_2_) while 100 µl of each diluted sample was loaded in separate cultured wells and carried out in triplicate. The cells were then examined under an inverted microscope to observe the cytotoxic effects of the tested materials on the MCF-7 cancer cell line. After applying 20 µl of MTT (BIO BASIC CANADA INC.) solution in each well and shaking the plate at 150 rpm for 5 minutes to fully mix the MTT reagent, the plate was incubated to allow the MTT to metabolize and produce purple formazan crystals. Following MTT incubation, the consumed medium was discarded, the formazan crystals were reconstituted in 200 µl of DMSO, and the plate was shaken at 150 rpm for five minutes. After subtracting the background absorbance at 620 nm, the optical densities (ODs) of the dissolved formazan crystals were measured spectrophotometrically at 560 nm. The concentration required to inhibit 50% of cell growth compared to untreated cells (IC₅₀) was used to express the results.

Cell viability (%) = $\frac{OD treated cells}{OD Untreated cells}\times100$

**Protein Denaturation Inhibition Test**

Screening for protein denaturation inhibitors is critical for studying anti-inflammatory effects. The protein denaturation test was performed with slight modifications of the procedure outlined by Gambhire et al. [28]. In the 5 mL reaction mixture, 200 µL of 1% BSA (Bovine serum albumin) solution was combined with 4.78 mL of PBS buffer (pH 7.4). The drug concentration was 10 µg/mL. The reaction mixture was placed into an incubator at 37 °C for 15 minutes, after which it was heated to 70 °C for 5 minutes. The reaction mixture was cooled, and the resulting turbidity was measured using a spectrophotometer set at 660 nm. The control consisted of phosphate buffer solution and BSA without the drug. The percentage of inhibition of denatured protein was calculated using the following equation:

Percentage of denaturation inhibition = 100 × (1-A2/A1).

Where A1 and A2 represent the absorbance values of the control sample and the tested drug, respectively.

**Figure S1. Representative flow cytometric DNA content histograms of MCF-7 breast cancer cells stained with Propidium Iodide (PI), displayed on a logarithmic fluorescence scale. Histograms are shown for (a) untreated control cells and (b) cells treated with the curcumin–glucosamine (CG) ligand at its IC₅₀ (7.51 ± 0.12 µg/mL) for 24 h. The logarithmic display is provided for comparison and facilitates visualization of the sub-G1 apoptotic population. Cell-cycle phase percentages (G0/G1, S, and G2/M) were determined from the corresponding linear fluorescence data using ModFit LT (Dean–Jett–Fox model), as presented in Figure 6.**

**BSA Binding Test :**

The nature of the interaction between newly synthesized compounds and proteins provides a new approach for the development of novel drugs. Since drugs are transported by albumin, it is important to study the interaction of new drugs with proteins [29]. The binding of drugs to serum albumin is a major contributor in understanding the interaction of drugs with body, as it affects the distribution, excretion, metabolism, and interaction with biological targets. Human albumin (HSA) in laboratory studies is often used in place of for bovine serum albumin (BSA) due to it is more available and lower cost. BSA has the same drug binding capacity because 76 % identity and 88 % amino acid sequence similarity with BSA. Through UV-Vis spectroscopy, the binding ability of the compound(s) is evaluated by observing changes in the characteristic absorption peak of bovine serum albumin (BSA) at 280 nm. The buffer, a phosphate-buffered saline (PBS) solution, contains 137.00 mM NaCl, 2.70 mM KCl, 10.00 mM Na₂HPO₄, and 1.80 mM K₂HPO₄. Curcumin complex solutions (100 mM/L) were prepared in grade water. Curcumin complex solutions (100 mM/L) were prepared in grade water, and BSA was dissolved in phosphate-buffered saline (20.00 mM/L). The BSA concentration was kept constant during the absorption experiments, while the complex concentration was increased from 10 µM to 100 µM. The mixture of 5.00 mL of each curcumin complex and BSA was incubated in the dark for 1 hour at room temperature with gentle shaking. The absorption spectra of the prepared solutions were recorded in the 200–500 nm range at room temperature using a Thermo Scientific Evolution™ Pro UV–Vis spectrophotometer (model 840–370400).

**Figure S2: 1% DNA gel electrophoresis of calf thymus DNA (CT-DNA) bound complex showing differences in migration and band intensity compared to free CT-DNA. Lane M represents unbound CT-DNA (50 µg/ml), while** **lanes 1-4 correspond to CT-DNA bound to the vanadyl complex at different concentrations (10-40 µg/ml). lanes 1’-4’ represent duplicate the same experiment foe lane 1-4. Lanes 5–6 and 5′–6′ represent duplicate experiments evaluating the DNA cleavage activity of the compound that were not included or discussed in the present manuscript and are shown only as part of the complete original gel image**

**Figure S3: Interaction between three compounds with the GLUT1 receptor. A: the complex, B: ligand only, and C: PNG ligand. Left: 2D interaction maps. Right: 3D ligand binding conformation**

**Table S1. Selected optimized bond lengths (Å) and bond angles (degrees) of the CG ligand and the [VO(CG)₂] chelate, calculated using the B3LYP/6-311G and B3LYP/LANL2DZ methods, respectively.**

| **Bond length (**Å**)** | **CG** | **[VO(CG)_2_]** | **Bond angles** | **CG** | **[VO(CG)_2_]** |
| --- | --- | --- | --- | --- | --- |
| **R(N14-C12)** | 1.29705 | 1.34815 | **A(N14-C12-C13)** | 115.790 | 123.069 |
| **R(C12-C13)** | 1.52320 | 1.43902 | **A(C12-C13-C15)** | 112.056 | 126.427 |
| **R(C13-C15)** | 1.53159 | 1.38619 | **A(C13-C15-O19)** | 119.913 | 120.913 |
| **R(C15-O19)** | 1.25138 | 1.35453 | **A(O19-C15-C16)** | 123.128 | 115.723 |
| **R(C15-C16)** | 1.46872 | 1.46650 | **A(O19-N14-O57)** | --- | 71.808 |
| **R(O19-V)** | --- | 1.90812 | **A(N14-O78-O19)** | --- | 56.930 |
| **R(N14-V)** | --- | 2.13253 | **A(O19-V-O57)** | --- | 121.201 |

**Table S2: Mean and SD values for MTT assay.**

| **Concentration (μg/ml)** | **CG ligand** | | **CG^+^ Quercetin** | | **[VO(CG)₂] Complex** | | **Quercetin** | |
| --- | --- | --- | --- | --- | --- | --- | --- | --- |
|  | **Mean** | **SD** | **Mean** | **SD** | **Mean** | **SD** | **Mean** | **SD** |
| 1000 | 3.48 | 0.00033 | 4.58 | 0.00166 | 3.66 | 0.00066 | 3.15 | 0.00066 |
| 500 | 3.30 | 0.00057 | 4.81 | 0.00057 | 5.13 | 0.00260 | 4.02 | 0.00057 |
| 250 | 3.85 | 0.00115 | 15.45 | 0.00284 | 35.76 | 0.00950 | 4.48 | 0.00088 |
| 125 | 4.40 | 0.00152 | 33.51 | 0.00698 | 70.51 | 0.00272 | 8.19 | 0.00145 |
| 62.5 | 7.56 | 0.00416 | 86.47 | 0.00751 | 91.47 | 0.00321 | 12.72 | 0.01225 |
| 31.25 | 50.57 | 0.00202 | 98.85 | 0.00145 | 99.26 | 0.00202 | 60.89 | 0.00115 |
| 0 | 100 |  | 100 |  | 100 |  | 100 |  |

**Table S3: Comparison of the ligand and its vanadyl complex with the EGFR receptor in terms of docking scores, RMSD_refine, and E_refine.**

| Compound | Docking score (kcal/mol) | Rmsd refine / (Å) | E_refine / (kcal/mol) |
| --- | --- | --- | --- |
| [VO(CG)_2_] | -4.3196 | 1.8387 | -20.1332 |
| CG | -4.9997 | 1.4738 | -15.2482 |

**Table S4:** **Comparison of the ligand and its vanadyl complex with the GLUT1 receptor in terms of docking scores, RMSD_refine, and E_refine.**

| Compound | Docking score / (kcal/mol) | Rmsd refine / (Å) | E_refine / (kcal/mol) |
| --- | --- | --- | --- |
| [VO(CG)_2_] | -10.2197 | 2.3825 | -18.7364 |
| CG | -6.8071 | 1.4714 | -17.3915 |
| PNG ligand | -5.4009 | 2.8899 | -14.3657 |

**Table S5: Table S6. Comparison of the ligand and the reference drug in ADMET toxicity parameters.**

| **Property** | **CG** | [VO(CG)_2_] | **Better?** |
| --- | --- | --- | --- |
| AMES toxicity | NO | NO | Equal |
| Max Tolerated Dose (Human) | 0.413 log mg/kg/day | 0.431 log mg/kg/day | Drug |
| hERG I Inhibition | NO | NO | Equal |
| hERG II Inhibition | NO | YES | Ligand |
| LD50 (Oral Rat Acute Toxicity) | 2.954 log mol/kg | 2.482 log mol/kg | Ligand |
| LOAEL (Chronic Toxicity) | 3.942log mg/kg_bw/day | 8.73 log mg/kg_bw/day | Drug |
| Hepatotoxicity | NO | NO | Equal |
| Skin Sensitisation | NO | NO | Equal |
| T.Pyriformis Toxicity | 0.285 log µg/L | 0.285 log µg/L | Equal |
| Minnow Toxicity | 2.818 log mM | 13.95 log mM | Drug |
